# ULBP1 Is Elevated in Human Hepatocellular Carcinoma and Predicts Outcome

**DOI:** 10.3389/fonc.2020.00971

**Published:** 2020-06-23

**Authors:** Nicholas J. W. Easom, Michael Marks, Dawda Jobe, Roopinder Gillmore, Tim Meyer, Mala K. Maini, Ramou Njie

**Affiliations:** ^1^Division of Infection and Immunity, University College London, London, United Kingdom; ^2^Hull University Teaching Hospitals NHS Trust, Castle Hill Hospital, Cottingham, United Kingdom; ^3^Faculty of Infectious and Tropical Diseases, London School of Hygiene and Tropical Medicine, London, United Kingdom; ^4^MRC Unit the Gambia at LSHTM, Fajara, Gambia; ^5^Royal Free Hospital, London, United Kingdom; ^6^Department of Oncology, UCL Cancer Institute, University College London, London, United Kingdom; ^7^Gambia Hepatitis Intervention Study (GHIS), IARC, Lyon, France

**Keywords:** hepatocellular carcinoma, biomarker, NKG2D, NK cell, liver

## Abstract

Hepatocellular carcinoma (HCC) remains a leading cause of cancer death worldwide, and despite recent immunotherapeutic advances there remains a need for improved diagnostic, prognostic, and therapeutic tools. UL-16 binding protein 1 (ULBP1) is a ligand of the activatory receptor Natural Killer cell Group 2 receptor D (NKG2D) and is found as a cell-surface protein on some malignant cells including on human hepatocellular carcinomas. We aimed to explore the biological and clinical significance of NKG2D ligands in the circulation of patients with HCC. We measured ULBP1 in the serum of two retrospective cohorts of patients with HCC from the PROLIFICA cohort in The Gambia (*n* = 43) and from a tertiary care setting in the UK (*n* = 72) by sandwich ELISA. Exosome isolation by size exclusion was used to compare ULBP1 concentration in exosomes and as free protein. Survival analysis was performed and multiple linear regression and Poisson regression were used to assess the independent effect of ULBP1 concentration. ULBP1 was raised in both cohorts with HCC regardless of the underlying liver disease, and was not associated with markers of cirrhosis such as platelet count or serum albumin. ULBP1 was present predominantly as free protein rather than bound to exosomes. Serum ULBP1 > 2000 pg/ml was associated with a significantly reduced survival in both cohorts (hazard ratios in Gambian and UK cohorts 2.37 and 2.1, respectively). The effect remained significant after adjustment for BCLC staging (*p* = 0.03). These data suggest that ULBP1 merits further investigation as a prognostic marker in HCC in diverse settings and should also be explored as a therapeutic target.

## Introduction

Hepatocellular carcinoma (HCC) is the third leading cause of cancer death worldwide (1), and prognosis remains poor even in rich countries, with limited treatment options (2). Worldwide, hepatitis B virus (HBV) is the most common underlying etiological agent (3), with hepatitis C and other liver diseases accounting for the remainder. HCC is relatively resistant to chemo- and radiotherapy, and although early-stage tumors can be cured by surgical resection or liver transplant, with radio-frequency ablation possible in a proportion of small tumors, most cases present late when treatment options are limited (2). The tyrosine kinase inhibitor Sorafenib prolongs survival but is not curative (4) and more recently PD-1 blockade with Nivolumab has been shown to induce objective response in a minority of patients (5). Despite this recent progress, novel treatment modalities are needed and immunotherapeutics involving NKG2D are one possible approach.

The NKG2D ligands ULBP1-6, MICA, and MICB are expressed by cells undergoing viral infection, malignant transformation and cellular stresses (6). They engage NKG2D, an activatory receptor expressed by NK cells and CD8 T cells, stimulating cytotoxicity (7). NKG2D has an important role in immune surveillance (8) and malignant tumors may release soluble NKG2D ligands as an immune evasion strategy to evade detection by NK and T cells and impair NKG2D function (9, 10). NKG2D ligands may be released by proteolytic shedding or on exosomes (11, 12). Soluble NKG2D ligands are generally thought to act by NKG2D downregulation, impairing the ability of NK and cytotoxic T cells to respond to target cells (9). However, in some murine systems soluble NKG2D ligands act differently, blocking a tonic NKG2D downregulation due to ligands expressed on the surface of parenchymal cells, resulting in improved NK cell killing (13).

The NKG2D pathway has received increasing attention over recent years as a potential therapeutic target in cancer (14, 15). Novel agents designed to target NK cells to NKG2D ligand-expressing tumor cells are under investigation, predominantly in the setting of hematological malignancy (16, 17). Recently antibodies against the α3 domain of MICA, simultaneously blocking the site of proteolytic cleavage and providing a second activatory signal to NK cells via the Fc-gamma receptor CD16, have been shown to be therapeutic in a mouse model of human melanoma (18), demonstrating that such approaches could be beneficial in solid-organ malignancy.

Liver tissue and HCC contain large numbers of NK and CD8 T cells. Immunohistochemical studies of HCC have demonstrated lymphocytic infiltrates with CD8 T cells predominating over CD4 cells (19, 20). More detailed characterization of lymphocytes in multiple studies of human liver has demonstrated populations of NK cells, T cells and NKT cells and more recently work from our group and others has demonstrated a population of liver-resident NK cells not represented in peripheral blood, and shown the presence of NK cells infiltrating HCC and demonstrating marked functional impairment (21–23). Single cell RNA-seq of cells isolated from HCC demonstrates multiple NK cell clusters and T cells exhibiting an exhausted phenotype (24, 25). Immunohistochemistry of tumor has shown NKG2D ligand loss at the cell surface associated with more poorly differentiated HCC and reduced disease-free survival (26). Given the limited treatment options in HCC and the availability of potential tools to recruit effector cells at the site of, and infiltrating the tumor, we therefore sought to explore whether NKG2D-ligands were shed into serum in patients with HCC, in what form, and whether they could have diagnostic or prognostic utility.

## Methods and Patients

### NKG2D Ligand ELISA

ELISAs for MICA, MICB, ULBP1, ULBP2, and ULBP3 (R&D) were performed as per the manufacturer's instructions. All samples and standards were assayed in duplicate. Plates were read immediately using an automated plate reader (Finstruments Multiskan) at 450 nm. Mean optical densities (OD) for each standard and sample were taken and antigen concentrations were calculated using Excel for Mac 2011 (Microsoft).

### Exosome Isolation and Imaging Cytometry

Exosomes were isolated from serum using Izon Science qEV Size Exclusion Columns according to the manufacturer's instructions. For identification of exosomes in serum, Fc receptor blocking reagent (Miltenyi) was added to paired serum, exosome and protein fractions before staining with BODIPY-FL (ThermoFisher) and ULBP1-PE (R&D). Samples were fixed with Cytofix (BD) before acquisition. Samples were acquired by ImagestreamXL (Amnis) and data analysis performed using IDEAS software (Amnis).

### PBMC Isolation

PBMC were isolated by density gradient centrifugation using Ficoll-Hypaque (GE Healthcare). Peripheral blood was collected into vacutainers containing EDTA (BD). Using 15 ml falcon tubes (Sarstedt), 10 ml blood was carefully layered onto 5 ml Ficoll-Hypaque and centrifuged at 2,000 rpm, acceleration 6, brake 4 in a Thermo Multifuge centrifuge. The PBMC layer was removed using a Pasteur pipette and washed in Roswell Park Memorial Institute (RPMI) medium (Gibco). Cells were either stained immediately for flow cytometry or counted using trypan blue (Sigma) and transferred to freezing medium (FBS (Sigma) with 10% dimethylsulfoxide (DMSO) (Sigma) at a concentration of 10 million cells/ml for cryopreservation, initially at−80°C before transfer to liquid nitrogen for long-term storage. Before use cells were thawed in a water bath at 37°C and washed in 20 ml RPMI.

### Flow Cytometry

All samples were treated with Fc receptor blocking reagent (Miltenyi Biotec) before staining. Surface staining was performed in 96 well plates (Sarstedt) in staining buffer of 50% PBS, 50% Brilliant Violet staining buffer (BD). Fixable live/dead stain (Life Technologies) was added to the staining buffer. Antibody staining was conducted for 15 min at 37°C in the dark before washing with PBS using the following monoclonal antibodies: CD3 PE-Cy7 and CD8 Alexa 700 (eBioscience), CD56 ECD (Beckman Coulter), NKG2D Alexa 488 (Biolegend). Cell viability was determined and dead cells excluded using fixable live/dead aqua stain (Invitrogen). Samples for were fixed in Cytofix (BD). Single fluorochrome compensation controls were made using compensation beads (BD). Compensation matrices were calculated initially in FACSDiva and edited where necessary in FlowJo X (TreeStar). Samples were acquired on LSR Fortessa (BD) and data analyzed in FlowJo X.

### Clinical Groups

Participants were recruited from the PROLIFICA (28) study at MRC Unit, The Gambia and from the Royal Free Hospital, London (demographic and clinical parameters in Table 1). The Gambian cohort was comprised of patients enrolled in the Prevention of Liver Fibrosis and Cancer in Africa (PROLIFICA) study. In the Gambian cohort, negative controls were hepatitis B surface antigen (HBsAg) negative with no liver mass on ultrasound, inactive CHB patients were HBsAg positive with HBV viral load <2000 IU/ml and ALT <80 IU/ml, active CHB patients were HBsAg positive with viral load >2,000 or ALT>80 IU/ml, CHB cirrhosis patients were HBsAg positive with a median liver stiffness by Fibroscan (Echosens) >10 kPa or cirrhosis on histology, HCC patients had HCC on histology or liver mass on ultrasound with alpha-fetoprotein >400 ng/ml. Patients referred to the liver clinic who had other tumors on imaging or histology (colorectal metastases, pancreatic tumor, cholangiocarcinoma, lymphoma, metastatic melanoma, sarcoma) were used as a comparator group. Serum was collected for ULBP1 measurement from untreated patients. Outcome data were collected by the PROLIFICA team and by the MRC Unit, The Gambia. Tumor volume was estimated by calculating the volumes of spheroids with dimensions measured by liver ultrasound scan, using the formula: volume = π^*^ ((D_1_+D_2_)/2)^3^/6.

**Table 1:** Clinical characteristics of the Gambian and UK HCC cohorts and Gambian control cohort.

**Parameter**	**Gambia HCC**	**UK HCC**	**Healthy control**
Male: Female ratio	32:11 (74.4%)	61:11 (84.7%) p = 0.27	6:17 (26.1%) p = 0.0004
Age (years)	41	66 (35–85) p <0.0001	54 (31–83) *p* = 0.017
HBsAg positive	40/43 (93%)	8/72 (11.1%) p <0.0001	0/23 (0%) p <0.0001
HCV RNA positive	4/36 (11.1%)	18/72 (25%) p = 0.15	0/23 (0%) p = 0.26
Bilirubin (μmol/L)	21 (2–341)	16 (6–65) p = 0.0045	12 (8–28) p = 0.0009
Albumin (g/dL)	32 (16–44)	40 (25–49) p <0.0001	44 (36–50) p <0.0001
WHO Performance	2 (0–5) (*n* = 24)	1 (0–3) p = 0.0002	-
AFP (ng/mL)	350 (350–18178) (n = 19)	35 (2–10,000) p = 0.087	-

*Median (range) for age, bilirubin, albumin of the Gambia HCC (n = 43), UK HCC (n = 72), and Gambia control (n = 23) cohorts, hepatocellular carcinoma (HCC) and healthy control groups. WHO performance status and AFP are shown for the full UK HCC cohort and a subset of the Gambia HCC cohort, n shown in table. Student's t-test with Welch's correction was used to calculate p values for continuous variables and Chi square with Yates' correction for categorical variables, using the Gambia HCC cohort as a reference*.

The UK cohort was derived from patients attending The Royal Free Hospital liver oncology clinic. Serum was collected for ULBP1 measurement from patients before any local, systemic or surgical treatment was received. HCC was defined as patients meeting radiological criteria for HCC on triple-phase CT or MRI or with features of HCC on histology following biopsy or resection. Patients were managed according to EASL-EORTC clinical practice guidelines 2012 (27). Outcome data were obtained using NHS Spine. For one experiment *ex vivo* human liver and HCC tissue provided by the Tissue Access for Patient Benefit service at the Royal Free Hospital was used as previously described (23).

### Statistical Analysis

The Mann Whitney-*U* test was used for comparisons of two unpaired groups, Spearman rank test was used for correlations of continuous variables. These tests were performed in GraphPad Prism version 6. *P* ≤ 0.05 was considered to be significant for all tests. Multiple linear regression and Poisson regression were performed in R (The R Project for Statistical Computing) version 3.4.2.

## Results

**Figure 1:**
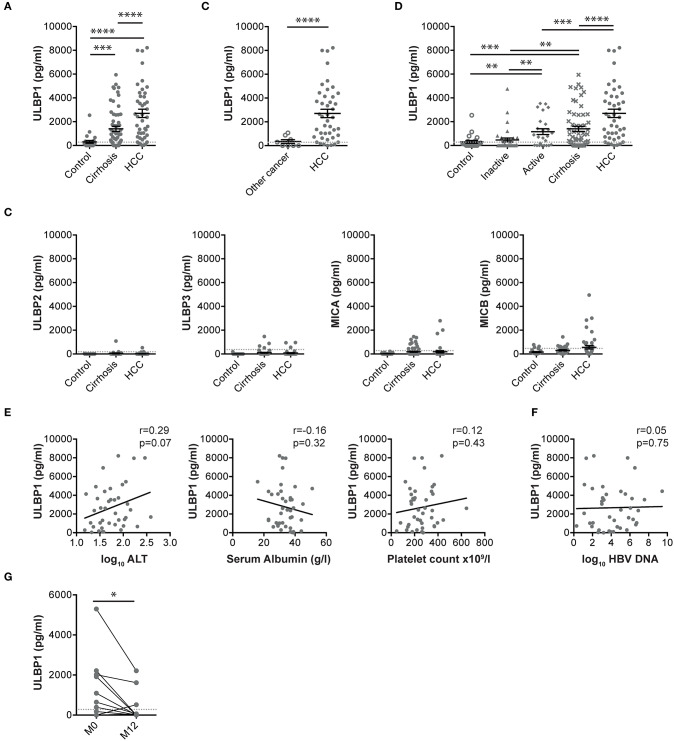
Serum ULBP1, but not other NKG2D ligands, is raised in patients with HCC in The Gambia. Serum ULBP1 measured by ELISA in patients with HCC (*n* = 43) in the Gambia compared with controls (*n* = 23) and patients with cirrhosis (*n* = 60, **A**) and other liver tumors (*n* = 8, **B**). Other NKG2D ligands in the same cohort **(C)**. Serum ULBP1 in Gambian patients with inactive CHB (*n* = 34), active CHB (*n* = 25), or CHB-associated cirrhotic liver disease, HCC and controls **(D)**. Serum ULBP1 concentrations in individuals with HCC against log10 serum ALT concentration (*n* = 40), serum albumin (*n* = 42), and platelet count (*n* = 43) **(E)** and against log10 HBV DNA concentration (*n* = 40, **F**). Paired serum ULBP1 in patients with HBV without HCC before and after 12 months of treatment with tenofovir (*n* = 50, **G**). Mean and SEM of all groups shown. Levels of significance: *p ≤ 0.05; **p ≤ 0.005; ***p ≤ 0.001; ****p ≤ 0.0001. Mann Whitney-*U* test was used for comparisons of two unpaired groups, Spearman rank test was used for correlations of continuous variables, Wilcoxon match-pairs signed rank test was used for comparisons of paired data.

Serum concentrations of the NKG2D ligand ULBP1 were elevated in Gambian patients with HCC compared with either patients with cirrhosis or with healthy controls (median ULBP1 concentrations 2626, 691, and 128 pg/ml, respectively, Figure 1A). A small cohort of individuals with non-HCC liver tumors had ULBP1 levels comparable to healthy controls (median 151 pg/ml) and significantly lower than those with HCC (Figure 1B). There was no change in the levels of the NKG2D ligands MICA, MICB, ULBP2, ULBP3 in these same cohorts (Figure 1C).

To examine whether elevated serum ULBP1 could be derived from diseased hepatocytes rather than exclusively HCC, we examined levels in patients with chronic hepatitis B (CHB), the most important underlying liver disease in The Gambia. Concentrations were higher in those with active or cirrhotic CHB than inactive carriers or controls, although remained significantly lower than in HBV-related HCC (Figure 1D). Serum ULBP1 was not associated with markers of liver disease including platelet count, alanine transaminase or serum albumin, or with HBV viral load (Figures 1E,F), suggesting that in the context of clinical HCC, ULBP1 production was independent of liver fibrosis, hepatocyte dysfunction and HBV replication. In 50 patients with HBV but without HCC treated with tenofovir according to EASL guidelines, ULBP1 was significantly reduced following 12 months of treatment compared with at treatment initiation (Figure 1G).

**Figure 2:**
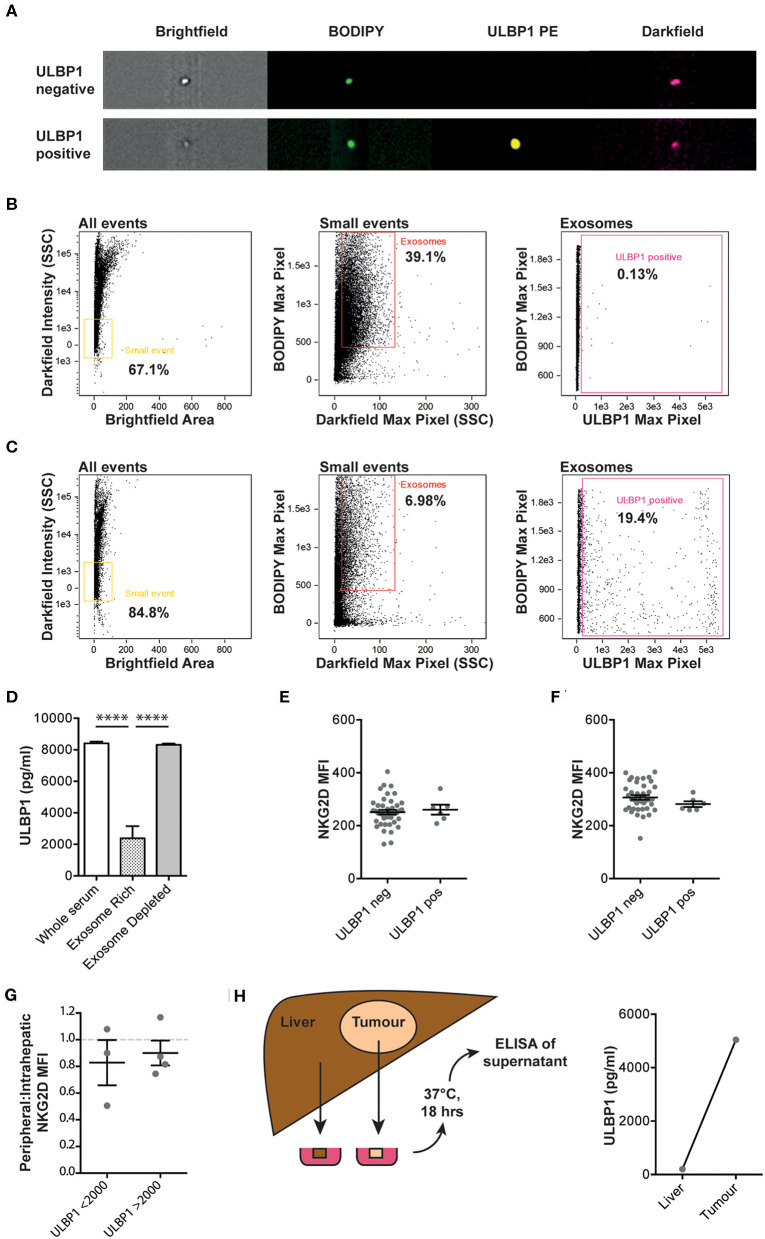
Imaging cytometry of serum from Gambian HCC patients with high levels of ULBP1, stained for lipid membrane (BODIPY) and ULBP1. Representative examples of ULBP1 negative and positive exosomes shown in **(A)**. Serum was separated into exosome and protein fractions by size exclusion column filtration and imaging cytometry of exosome-rich **(B)** and exosome depleted **(C)** fractions was performed. Exosomes gated as small, BODIPY positive events, a proportion of which are ULBP1 positive. ULBP1 concentration of whole serum, exosome rich and exosome depleted fractions was assessed by ELISA as in Figure 1 (**D**, *n* = 16). Flow cytometry of cell-surface expression of NKG2D on peripheral blood NK cells **(E)** and CD8 T cells **(F)** in a subset of Gambian patients with HCC or HBV. Ratio of NKG2D MFIs on paired peripheral and intrahepatic NK cells stratified by serum ULBP1 (**G**, *n* = 7). Cartoon depicting resection of tumour and liver tissue for short-term culture before ULBP1 ELISA of the conditioned media, ULBP1 concentration by ELISA of conditioned media from paired liver and tumour tissue (*n* = 1, **H**). Mean and SEM of all groups shown. Levels of significance: *p ≤ 0.05; **p ≤ 0.005; ***p ≤ 0.001; ****p ≤ 0.0001. Mann Whitney-*U* test was used for comparisons of two unpaired groups.

To understand whether ULBP1 was released on exosomes rather than as free protein, size exclusion chromatography was used to separate patient serum into an exosome-rich fraction and an exosome-depleted fraction. Imaging cytometry was used to identify individual exosomes by size and uptake of BODIPY membrane stain. Representative examples of ULBP1 positive and negative exosomes are shown in Figure 2A. The exosome-rich fraction contained many more small, BODIPY-positive events than the exosome-depleted fraction (39.1 vs. 6.98%) and very few ULBP1 positive exosomes were seen in the exosome-rich fraction (Figures 2B,C), suggesting that this was not the major form in which ULBP1 was present in serum. When split into exosome-rich and exosome-depleted fractions the ULBP1 concentration of serum and exosome-depleted fractions was similar, whereas the ULBP1 concentration of the exosome-rich fraction was significantly reduced (Figure 2D).

In a subset of Gambian patients with CHB or HCC and where PBMCs were available, NKG2D expression on peripheral blood NK cells and T cells was measured by flow cytometry to test whether it was downregulated by the presence of circulating NKG2D-ligands. The expression of NKG2D on the surface of NK and T cells within this patient cohort was not altered in those patients where ULBP1 was detectable in serum compared to those with no detectable ULBP1 (Figures 2E,F, gating strategy shown in Supplementary Figure 1). In seven participants from the Gambian HCC cohort we were able to compare NKG2D expression between peripheral and intrahepatic NK cells. There was no difference in the ratio of peripheral:intrahepatic NKG2D expression between individuals with ULBP1 above or below 2000 pg/ml (Figure 2G). In one individual from the UK HCC cohort, small fragments of liver and tumor were weighed and incubated with a volume of complete RPMI in μl equivalent to 10x the weight of the tissue in μg. The tissue was incubated for 18 h at 37**°**C, the supernatant was removed and ULBP1 ELISA was performed. The ULBP1 concentration in the tumor-conditioned media reached a similar high level to that found in the sera of HCC patients (5041 pg/ml), whereas the concentration in the conditioned media from surrounding liver was negligible (202 pg/ml). These data reveal the capacity, at least in this individual, of HCC (and/or its immune milieu) to shed ULBP1 as a putative immune evasion strategy.

**Figure 3:**
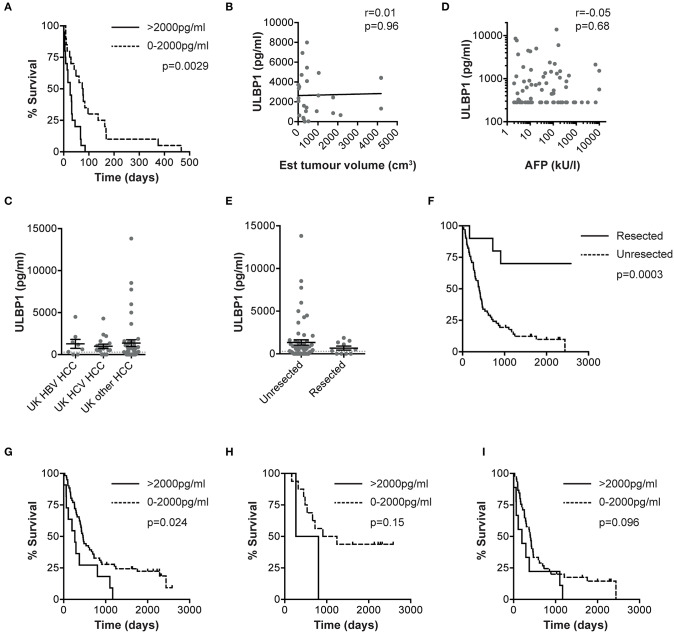
ULBP1 predicts outcome in HCC patients from the The Gambia and The UK. Kaplan-Meier curve comparing survival from the day of baseline clinical assessment between groups with serum ULBP1 above and below 2000 pg/ml in The Gambia (*n* = 37, **A**). Serum ULBP1 concentrations in individuals with HCC against estimated tumor volume (*n* = 27, **B**). Serum ULBP1 concentration in HBV-associated HCC (*n* = 8), HCV-associated HCC (*n* = 18) and HCC of other causes (*n* = 45), from a UK cohort **(C)**. Serum ULBP1 concentration in UK patients against alpha fetoprotein (AFP, **D**). Serum ULBP1 concentration **(E)** and Kaplan-Meier curve of survival **(F)** in HCC patients that went on to resection (*n* = 10) and those whose tumors were not resected (*n* = 61). Kaplan-Meier curve comparing survival from the day of baseline clinical assessment between groups with serum ULBP1 above and below 2000 pg/ml in the UK whole cohort (*n* = 72, **G**). Kaplan-Meier curves comparing survival between groups with serum ULBP1 above and below 2000 pg/ml in UK patients with early stage (BCLC stage 0/A, *n* = 18, **H**) and late stage (BCLC B-D, *n* = 54, **I**) HCC. Mean and SEM of all groups shown. Levels of significance: *p ≤ 0.05; **p ≤ 0.005; ***p ≤ 0.001; ****p ≤ 0.0001. Mann Whitney-*U* test was used for comparisons of two unpaired groups, Spearman rank test was used for correlations of continuous variables. For Kaplan-Meier curves, *p*-values for difference in survival and hazard ratios were calculated by log-rank test.

We next sought to examine whether ULBP1 levels in serum could predict outcome in HCC. In a subset of Gambian patients where retrospective follow-up data was available, there was significantly shorter survival in HCC patients with serum ULBP1 >2000 pg/ml at the time of presentation (median survival 26 vs. 74 days, *p* = 0.0029, hazard ratio (HR) 2.37, Figure 3A). Median survival for the whole cohort was 33 days from blood sampling to death. This was not simply a reflection of high serum levels of ULBP1 reflecting larger tumors since ULPB1 levels did not correlate with HCC volume estimated by ultrasound scan (Figure 3B).

Using a second, larger (*n* = 72) cohort of HCC patients we sought to validate the use of ULPB1 as a prognostic marker for HCC. In this UK-based cohort with HCC arising on a background of a range of underlying liver pathologies we were also able to examine whether elevation of ULBP1 was restricted to HBV-related HCC. We found that ULPB1 was similarly elevated in HCC resulting from HBV, HCV or other etiologies (median 907, 636, 301 pg/ml for HBV/HCV/other causes, respectively, differences not significant, Figure 3C). There was no association between serum ULBP1 and AFP (alpha fetoprotein), suggesting that ULBP1 secretion is mechanistically distinct from AFP production (Figure 3D). Importantly there was no significant difference in ULBP1 concentration between resected and unresected tumors at a population level (Figure 3E), and no significant difference in proportions with ULBP1 concentrations >2000 pg/ml (11/61 vs. 0/10, *p* = 0.34 by Fisher's exact test). As expected, tumor resection was associated with a significant improvement in survival compared with unresected HCC (HR 0.16, *p* = 0.0003, Figure 3F). In a retrospective analysis of outcomes, serum ULBP1 concentration >2000 pg/ml was again significantly associated with a poorer prognosis (median survival 267 vs. 462 days, *p* = 0.024, HR 2.1, Figure 3G). Median survival for the whole cohort was 427 days from blood sampling to death.

When divided into early (Barcelona Clinic Liver Cancer (BCLC) 0/A, Figure 3H) and late (BCLC B/C/D, Figure 3I) stage disease, survival differences according to ULPB1 levels in both groups showed raised hazard ratios for death, suggesting ULBP1 may be prognostic in both early and late disease, although p values were non-significant owing to a lack of statistical power (median survival 1077 vs. 535 days, *p* = 0.15, HR = 2.9 in early stage, 399 vs. 198 days, *p* = 0.096, HR = 1.8 in late stage). In univariable analysis, ULBP1 had a HR for death of 2.59 (*p* = 0.004, Table 2). Raised AFP was not significantly associated with worse prognosis, in keeping with previous work showing very limited prognostic effect even in large cohorts (29). BCLC and CLIP (Cancer of the Liver Italian Program (30)) scores were predictive of outcome, WHO performance status did not reach significance (*p* = 0.063). Importantly, in multivariate analysis including BCLC stage (which is used to guide clinical management (27)), age and sex, ULBP1 was an independent predictor of survival (hazard ratio 2.11, 95% CI 1.02-4.02, Table 3). Similar data incorporating CLIP score is shown in Supplementary Table 1.

**Table 2:** Univariable analysis of risk factors for mortality in UK HCC cohort.

**Variable**	**Unadjusted HR**	**95% CI**	***p*-value**
Age (continuous variable)	1	0.98–1.03	0.49
Male	1.96	0.95–4.73	0.09
ULBP1 > 2000 pg/ml	2.59	1.28–4.80	0.004
AFP 20-200	1.34	0.73–2.41	
AFP >200	1.89	0.96–3.56	0.1692
BCLC 0	1	–	
BCLC A	1.48	0.38–9.68	
BCLC B	4.64	1.31–29.47	
BCLC C	3.89	1.17–24.12	
BCLC D	3.13	0.68–21.88	0.009
WHO Performance 1	1	–	
WHO Performance 1	1.46	0.84–2.52	
WHO Performance 2	6.73	1.61–19.06	
WHO Performance 3	1.1	0.32–2.80	0.063
CLIP 0	1	–	
CLIP 1	2.09	1.08–4.11	
CLIP 2	3.61	1.77–7.34	
CLIP 3	8.99	3.22–21.85	
CLIP 4	21.39	3.38–75.16	0.006

*Hazard rate ratio for death and p value calculated by linear regression for ULBP1, AFP as a categorical variable, and three clinical scoring systems BCLC stage, WHO performance status and CLIP score. For AFP, BCLC, WHO performance and CLIP p values for trend are shown*.

**Table 3:** Multivariate analysis of risk factors for mortality in UK HCC cohort using age, sex, and BCLC group.

**Variable**	**Unadjusted HR**	**95% CI**	***p*-value**	**Adjusted HR**	**95% CI**	***p*-value**
Age (continuous variable)	1	0.98–1.03	0.49	0.99	0.97–1.03	0.99
Male	1.96	0.95–4.73	0.09	1.31	0.61–3.26	0.52
ULBP1 > 2000 pg/ml	2.59	1.28–4.80	0.004	2.11	1.02–4.02	0.03
BCLC A	1.48	0.38–9.68	0.62	1.38	0.35–9.12	0.68
BCLC B	4.64	1.31–29.47	0.04	3.61	0.95–23.71	0.1
BCLC C	3.89	1.17–24.12	0.06	3.44	1.00–21.65	0.1
BCLC D	3.13	0.68–21.88	0.17	2.81	0.59–20.09	0.22

*Age, sex, BCLC group, and serum ULBP1 >2000 pg/ml as a categorical variable were included in a Poisson regression model to calculate multivariate hazard rate ratios for death and associated p values*.

## Discussion

Shed NKG2D ligands have been shown to impair NK and T cell function *in vitro* (9, 31) and to mediate tumor evasion in animal models (32, 33), although in some settings soluble NKG2D ligands may paradoxically mediate tumor rejection (13). In studies of HCC, soluble MICA expression has been associated with poor prognosis (34); conversely, expression of ULBP1 on the tumor surface has been associated with improved survival (26). We did not demonstrate elevation of MICA in patients from The Gambia, but found elevated serum ULBP1, suggesting the loss of cell surface ULBP1 seen in other studies may be due to shedding of this ligand by HCC into the circulation. Immunohistochemical staining of tumor tissue for ULBP1 combined with serum measurement of ULBP1 may be able to demonstrate a correlation between ULBP1 loss at the cell surface and ULBP1 presence in serum. In addition it will be revealing to understand whether other histological features of the tumor including histological subtype, vascularity and immune infiltration are associated with ULBP1 secretion as these may provide insights into the mechanism of ULBP1 production and shedding. Elevated MICA levels in HCC have been shown to be highly correlated with vascular invasion (34). As ULBP1 was not predominantly present on membrane-bound vesicles, shedding may occur by the action of a protease as previously described for other NKG2D ligands (35), although in some *in-vitro* systems ULBP1 is shed only at low levels (12). NKG2D ligands are known to be differentially expressed in a variety of malignant settings and to behave non-redundantly (36), which may explain why the other NKG2D ligands were not elevated. NKG2D was not downregulated on peripheral NK or CD8 T cells in the context of detectable ULBP1, but may have been affected within the tumor micro-environment, where NKG2D is known to be significantly downregulated (23). Future work should combine measurement of serum ULBP1 with phenotypic and functional assessment of tumor-infiltrating NK cells.

We have demonstrated that soluble ULBP1 is secreted in the context of HCC, but not metastases of other tumors to the liver, revealing a degree of diagnostic specificity. Although serum ULBP1 was significantly higher in patients with HCC, the fact that is was also detectable to a variable degree in HBV-related disease without HCC makes it less appealing as an HCC screening tool. Instead, our data show that ULBP1 elevated above 2000 pg/ml is a predictor of poor prognosis in HCC, pointing to the need to explore its utility as a prognostic biomarker in larger studies. Although our test and validation cohorts were small, ULBP1 showed consistent predictive capacity in patients from the UK and the Gambia, despite differences in etiology and overall survival between cohorts, and the management options available. The observation that serum ULBP1 falls in patients with active HBV following one year of HBV treatment with the nucleotide reverse-transcriptase inhibitor tenofovir, coupled with the numerically greater (but not statistically significant) ULBP1 in HBV-associated HCC than in HCC without HBV or HCV suggests a direct effect of HBV on ULBP1 production. The mechanism of this is yet to be elucidated, it may be caused by DNA damage or clonal expansion of hepatocytes (37), or another action of HBV. Treatment of HBV with tenofovir is known to reduce the incidence of HCC (38, 39). This raises the question of whether ULBP1 could predict progression to HCC. Whether ULBP1 can predict progression to HCC and the possibility of incorporating this marker into HCC screening should be examined in a large, prospectively followed and well characterized clinical cohort.

The UK HCC cohort was older, had less severe liver disease, less HBV and more HCV compared with the Gambian HCC patients. In the Gambia diagnosis was by ultrasound and AFP with histopathology in some cases and no specific management was available, whereas patients in the UK cohort were diagnosed using triple phase CT supported by MRI and histopathology where appropriate and managed with liver resection, liver transplant, trans-arterial chemo-embolisation and systemic chemotherapy according to EASL-EORTC guidelines (27). Triple-phase CT and MRI imaging were unavailable in both the study and the local health service in The Gambia and it would have been unethical to try to provide this given the absence of treatment options. We expect that in an endemic area our diagnostic strategy would have led to more under-diagnosis (and exclusion from the study) than over-diagnosis. The two settings are complementary—one reflects the resource-limited setting where the majority of HCC occurs and where a serum-based test might contribute greatly to the available diagnostic tools, the other provides a high level of clinical characterization to validate the findings and represents the sort of cohort in which novel immunotherapy could be trialed. It is interesting that the hazard ratios for death were of a similar magnitude in the two cohorts despite the large difference in survival. Median survival in the Gambian cohort was 33 days compared with 427 days in the UK cohort, hazard ratios for death associated with a ULBP1 > 2000 pg/ml were 2.4 and 2.1, respectively. The study was not designed to examine the reasons for this difference, which is likely multifactorial. It may combine late diagnosis in The Gambia compared with the UK where at-risk patients are screened regularly, improved survival following diagnosis due to treatment or other factors, or differences in the pathology in the different settings. This is supported by the difference in WHO performance status at enrolment between the UK and Gambian HCC cohorts (0.69 vs. 1.958, *p* = 0.0002, Table 1). However the consistency of our findings suggests ULBP1 is prognostic in a variety of settings and implies that the effect of ULBP1 may be similar in early and late stage HCC. Elevated ULBP1 predicted poor outcome even after accounting for BCLC stage and was unrelated to AFP, suggesting that ULBP1 may capture additional information about tumor biology distinct from that of existing clinical and biochemical markers and supports the idea that NKG2D ligand secretion can promote tumor progression *in vivo*.

Further work is required to confirm the findings in a large, prospective cohort, where appropriate clinical cut-offs for ULBP1 can be explored. Follow-up of the existing HBV cohort will allow us to assess whether elevated ULBP1 levels may predict the development (as well as the outcome) of malignancy in the setting of HBV-related liver disease. Future studies, possibly using humanized mouse models, are required to explore whether the ULBP1/NKG2D system could be a novel therapeutic target in HCC. In a recent study using a murine model, antibodies blocking MICA secretion by tumors while simultaneously activating NK cells were shown to be therapeutic in a model of melanoma (18). Our data support the design and evaluation of a similar antibody to bind ULBP1 in human HCC.

## Data Availability Statement

The datasets generated for this study are available on request to the corresponding author.

## Ethics Statement

Studies were conducted in accordance with the declaration of Helsinki and informed consent was obtained from all subjects. Blood sampling in Gambian patients was approved by the joint Gambia Government-MRC Gambia Ethics Committee, The Gambia as SCC1379 and SCC1266. Blood and tissue sampling of UK patients was approved by the University College London-Royal Free Hospital Research Ethics Committee, ref no. 10/H0720/34 (blood), 11/WA/0077 (liver resections).

## Author Contributions

NE, MKM, and RN: conceptualization. NE: data curation, visualization and project administration. NE and MM: formal analysis. NE, MM, MKM, and RN: funding acquisition. NE and DJ: investigation. NE, MM, RG, MKM, and RN: methodology. NE, DJ, RG, and TM: resources. MM: software. MKM, RN, and RG: supervision. NE and MKM: validation and writing-original draft. NE, MM, DJ, RG, TM, MKM, and RN: writing-review and editing. All authors contributed to the article and approved the submitted version.

## Conflict of Interest

MKM has received laboratory research funding unrelated to this study from Roche, Gilead and Immunocore, and sat on advisory boards for Roche, Gilead, Janssen and Arbutus. TM has served as a consultant for Bristol-Myers Squibb, Bayer, Ipsen, and Eisai. The remaining authors declare that the research was conducted in the absence of any commercial or financial relationships that could be construed as a potential conflict of interest.
