# Supplementary material for: ULBP1 Is Elevated in Human Hepatocellular Carcinoma and Predicts Outcome
Source: Front Oncol. 2020 Jun 23;10:971. doi: 10.3389/fonc.2020.00971 (PMC7324784; doi:10.3389/fonc.2020.00971)
Supplement: Supplementary file 1 [file Table_1.pdf]

Supplementary Table 1: Multivariate analysis of risk factors for mortality in UK HCC cohort using age, sex and CLIP score

Variable	Unadjusted HR	95% CI	p-value	Adjusted HR	95% CI	p-value
**Age (continuous variable)**	1	0.98 - 1.03	0.49	1.012	0.989 - 1.04	0.25
**Male**	1.96	0.95 - 4.73	0.09	1.58	0.74 - 3.91	0.27
**ULBP1 > 2000pg/ml**	2.59	1.28 - 4.80	0.004	2.12	0.96 - 4.32	0.048
**CLIP 1**	2.09	1.08 - 4.11	0.03	1.62	0.79 - 3.31	0.18
**CLIP 2**	3.61	1.77 - 7.34	0.00035	3.34	1.61 - 6.90	0.001
**CLIP 3**	8.99	3.22 - 21.85	0.000005	8.04	2.86 -19.82	0.0002
**CLIP 4**	21.39	3.38 - 75.16	0.00004	21.61	3.37 - 78.46	0.00006

Age, sex, CLIP score and serum ULBP1 >2000pg/ml as a categorical variable were included in a Poisson regression model to calculate multivariate hazard rate ratios for death and associated p values.
