# Supplementary figures and images for: ULBP1 Is Elevated in Human Hepatocellular Carcinoma and Predicts Outcome
Source: Front Oncol. 2020 Jun 23;10:971. doi: 10.3389/fonc.2020.00971 (PMC7324784; doi:10.3389/fonc.2020.00971)

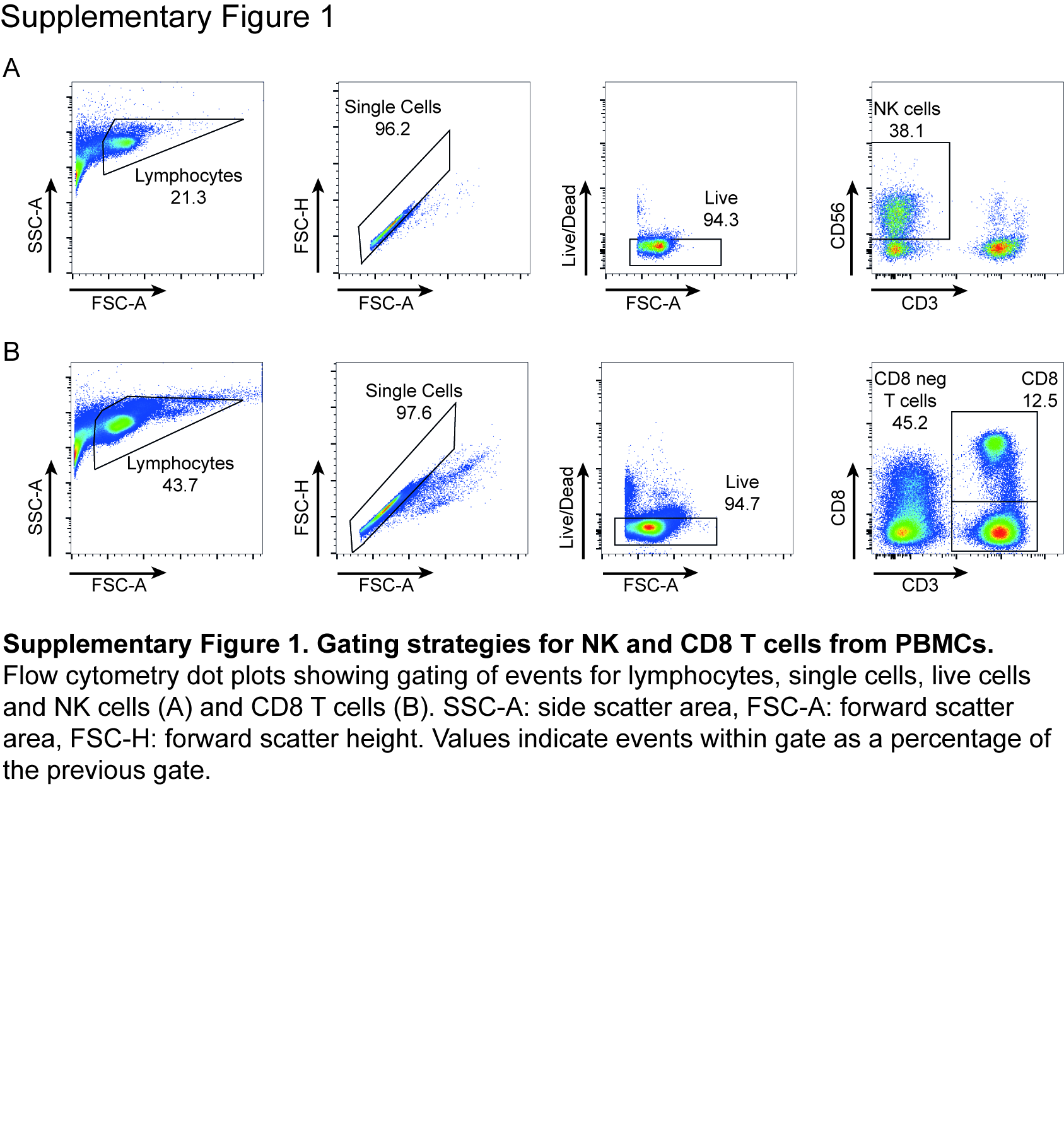

Supplement: Supplementary file 2 [file Image_1.TIF]
